# Physicochemical properties, oxidative stability and antioxidant capacity of clean label meat-based sauces: effects of phenolic extracts addition and cold storage

**DOI:** 10.1007/s13197-020-04519-x

**Published:** 2020-05-20

**Authors:** Grażyna Bortnowska, Sylwia Przybylska, Robert Iwański

**Affiliations:** Faculty of Food Sciences and Fisheries, West Pomeranian University of Technology in Szczecin, ul. Papieża Pawła VI, 3, 71-459 Szczecin, Poland

**Keywords:** Meat sauces, Native starch, Natural antioxidants, Chicken meat, Antioxidant capacity, Rheological properties

## Abstract

Physicochemical properties, oxidative stability and antioxidant capacity (AC) of clean label meat-based sauces (MBSs) were investigated with reference to cold storage time (ST) and addition of phenolic extracts (PEs): green coffee bean (GCE), green tea (GTE), knotweed rhizome (KRE). All parameters determined were compared to a control sample (CS), prepared without extracts. MBSs enriched with GCE or KRE were stable during the whole ST (90 days), while the CS and samples containing GTE, showed descending trend regarding physical stability after 10 days of storage. MBSs prepared with PEs (90 days storage) demonstrated: peroxide value (PV) < 8.5 mEq O_2_/kg lipids, TBARS < 5.6 mg MDA/kg lipids, carbonyl content < 4.7 nmol/mg protein, and the values were significantly (*p* < 0.05) smaller than those found in CS. Irrespectively of the applied measurements (ABTS, DPPH, FRAP) the values of AC (trolox equivalent, TE) decreased with ST, and regarding ABTS assay were in ranges: 0.73–0.42 mM TE (CS), 3.54–2.75 mM TE (GCE), 4.89–3.29 mM TE (GTE) and 3.86–2.25 mM TE (KRE). ANOVA revealed that rheological parameters obtained from Herschel–Bulkley′s and Bohlin′s models were predominantly (*p* < 0.001) affected by ST. After 90 days of storage, values of the desirability index were significantly (*p* < 0.05) higher for GCE-fortified MBS than for samples prepared with GTE and KRE. The results of this study can be useful to optimize composition of meat-based sauces containing bioactive ingredients and exhibiting desired by the consumer sensory features.

## Introduction

Commercially produced emulsion sauces (ESs) belong to ready-to-eat food and are added to many products, mainly to increase their sensory qualities. In recent years, along with the increase in nutritional awareness, consumers are looking for clean label sauces characterized also by: low fat content, increased nutritional value, and the presence of ingredients that can reduce the risk of many diseases (Bortnowska et al. 2013; Nascimento et al. 2018).

The main components of ESs are: vegetable oils, emulsifiers and hydrocolloids, used as thickeners and gelling agents. Meat is an excellent source of proteins that form compact coatings around oil droplets and exhibit satisfactory antioxidant capacity, moreover it contains many bioactive ingredients, including vitamins and minerals. Experimental studies revealed that waxy maize starch exhibits many specific attributes, because it: swells quickly, gives sticky texture, hardly retrograde and can be easily digested (Bortnowska et al. 2013). Sauces prepared with the use of meat relatively quickly undergo spoilage because of: microbial, chemical or meat endogenous enzymes actions. Particularly, lipids and proteins are very susceptible to oxidation that may occur during processing and storage. Lipid hydroperoxides have been identified as primary products of autoxidation and their decomposition, yields secondary oxidation products, e.g. aldehydes and ketones (Cheng 2016). The main oxidative modifications of proteins takes place at the side chains of amino acids, which include: sulfhydryl groups oxidation and carbonyl groups formation (Diao et al. 2016).

In order to prolong the shelf life of food products a number of thermal and non-thermal methods, including ultrasound technique, are used (Espitia et al. 2014; Zinoviadou et al. 2015; Al-Hijazeen et al. 2016; De Souza et al. 2017). For the preserving of food prepared with the application of raw meat, thermal methods (e.g. pasteurization) are very useful, because flavor of the finished product is shaped as a result of taste products and aroma compounds generation (Jongberg et al. 2013). Among natural food preservatives, phenolic compounds (PCs) gain considerable attention, by reason that they exhibit: antioxidative and antimicrobial properties as well as retain color in meat products during storage time. Moreover it should be underlined, that PCs demonstrate hepatoprotective, anti-inflammatory, and anticancer effects (Galanakis 2018; Yildirim-Elikoglu and Erdem 2017). PCs represent a large and diverse group of substances, such as: phenolic acids, flavonoids and stilbenes. Antioxidant activity of PCs is associated with their ability to: chelate transition metals, neutralize free radicals through hydrogen atom transfer (HAT) or single electron transfer (SET) and others (Liang and Kitts 2014). Resveratrol (3,4′,5-trihydroxystilbene) was found in many plant species, e.g. in Japanese knotweed. This compound exists as cis- and trans-isomers, however trans-isomer appears to be more biologically active and stable natural form (Gülçin 2010). The major PCs extracted from green coffee beans are chlorogenic acids with the most abundant 5-O-caffeoylquinic acid. Green tea extracts are composed mainly of catechins, between them (-)-epigallocatechin gallate is regarded to be the most important because of its high content and excellent bioactivity (Roginsky and Alegria 2005). Review of the available literature showed that no study concerned the use of commercially available extracts containing PCs belonging to different classes to prevent chemical and physical alterations of sauces prepared with fresh meat and low amylose starch.

In this context, the aim of this work was to examine the impact of both phenolic extracts addition and cold storage on oxidative stability, antioxidant capacity and physicochemical characteristics of clean label meat-based sauces.

## Materials and methods

### Materials

Chicken meat (*M. pectoralis superficialis*) (22.4 wt% protein, 1.8 wt% fat, 74.2 wt% moisture, pH 5.63) was purchased from local meat purveyor. Rapeseed oil (7 wt% saturated, 65 wt% monounsaturated and 28 wt% polyunsaturated fatty acids) was bought from local supermarket. Native waxy maize starch (0.54 wt% amylose, 9.62 wt% moisture, 0.39 wt% proteins) was donated by Ingredion GmbH (Germany). Commercial extracts of: green coffee bean [*Coffea robusta* (L.)], 52.5 wt% polyphenols (47.4 wt% chlorogenic acids including 12.6 wt% 5-O-caffeoylquinic acid; green tea [*Camellia sinensis* (L.) O. Kuntze], 41.3 wt% polyphenols (20.9 wt% catechins including 11.2 wt% (-)-epigallocatechin gallate) and knotweed rhizome (*Reynoutria* Houtt., *Polygonaceae*) (99 wt% trans-resveratrol) were donated by Eusa Colors (Poland). 6-hydroxy-2,5,7, 8-tetramethylchroman-2-carboxylic acid (trolox), 2,2′-azino-bis(3-ethylbenzothiazoline-6-sulfonic acid) diammonium salt (ABTS), 2,2-diphenyl-1-picrylhydrazyl (DPPH), 2-thiobarbituric acid (TBA), 2,4-dinitrophenylhydrazine (2,4-DNPH), 2,4,6-tris (2-pyridyl)-s-triazine (TPTZ) and other chemicals were purchased from Merck (Poland).

### Sauces preparation

Meat homogenate (MH) was produced by homogenizing (90 s, 14,000 rpm) ground chicken meat with salt solution (0.4 M, pH 6.8, 4 °C). Waxy maize starch (WMS) was dispersed in distilled water and heated under agitation in water bath up to 95 °C for 20 min. To the MH slowly rapeseed oil was incorporated and the mixture was stirred for 10 min with a K45SS kitchen robot (KitchenAid Inc., St. Joseph Michigan, USA). Then, under stirring: green coffee extract (GCE), green tea extract (GTE) or ethanolic solution of knotweed rhizome extract (KRE) and gelatinized WMS (22 °C) were added. The pH of meat-based sauces (MBSs) was adjusted to 4.0 using acetic acid. Finally, the MBSs contained: 30 wt% rapeseed oil, 3.5 wt% chicken meat, 4.0 wt% WMS and 0.1 wt% of: GCE, GTE or KRE in relation to phenolic compounds (PCs) content. The control sample (CS) was prepared without extracts addition. MBSs were placed into 200 mL glass jars, closed using twist-off closures and heated in a water bath at 85 °C for 20 min (time estimated experimentally) and then subjected to storage (4 °C). Analyses were performed on day: 1, 10, 30, 60 and 90. All experiments were performed at 4 °C unless otherwise stated.

### Physicochemical properties determination

Physical stability of MBSs was monitored by measuring the extent of gravitational phase separation and using centrifugation assay. The stability index (SI) was calculated from equation: SI (%) = (HC/HT) × 100, where: HC, height of creamed layer and HT, initial height of MBSs. Rheological properties were measured using a strain/stress controlled AR-G2 rheometer (TA Instruments, New Castle, DE, USA), equipped with a cone-plate geometry (2° cone angle, 60 mm diameter, 62 µm gap). Flow curves were determined in the range of shear rate from 0.01 to 100 s^–1^ and thus obtained data were fitted to the Herschel–Bulkley’s model: $\upsigma =\upsigma _{0} + k{\dot{\upgamma}} ^{{\text{n}}}$, where: σ, shear stress (Pa); σ_0_, yield stress (Pa); k, consistency coefficient (Pa s^n^); ${\dot{\gamma }}$, shear rate (s^−1^) and n, flow behavior index (–). Oscillatory tests (0.1 − 10 Hz) were conducted inside the linear viscoelastic region: storage modulus (G′, Pa), loss modulus (G′′, Pa) and complex modulus (G*, Pa) were recorded versus frequency (ω). Bohlin’s parameters were assessed from the equation: G* = A_B_ ω^1/z^, where: z, coordination number (dimensionless) and A_B_, proportional coefficient (Pa s^1/z^). Sauter mean diameter: D[3,2] = ∑n_i_d_i_^3^/∑n_i_d_i_^2^, where: n_i_, number of the particles with diameter d_i_, was determined according to Caporaso et al. (2016) using a Mastersizer 2000 (Malvern Instruments, UK). The coalescence index (CI) was calculated from the equation: CI = (D[3,2]_t+SDS_ − D[3,2]_in+SDS_)/D[3,2]_in+SDS_, where: D[3,2]_t+SDS_ and D[3,2]_in+SDS_, the values of D[3,2] in the presence of SDS (s*odium dodecyl sulfate)* at any time of storage (t) and initial value (in), respectively. Color measurements were made with a HunterLab Digital Color Difference Meter, type D25-2A (Hunter Associates Laboratory Inc., Fairfax, VA, USA). Experimental data were expressed as: chroma, C* = (a*^2^ + b*^2^)^1/2^) and hue angle, H^°^ = tan^–1^ (b*/a*), where: a* (–a*, greenness; + a*, redness), and b* (–b*, blueness, + b*, yellowness).

### Free fatty acids and lipid oxidation

Free fatty acids (FFA) content (% oleic acid) was determined according to AOAC Official Method 940.^28^ (1999). The peroxide value (PV) was measured in milliequivalents of peroxide per kg lipids (mEq O_2_/kg lipids), according to ISO (2017). Thiobarbituric acid reactive substances (TBARS) were determined by the method of Caporaso et al. (2016) and expressed as mg malondialdehyde (MDA)/kg lipids.

### Carbonyl content, total sulfhydryl content, surface protein concentration

Protein carbonyl and total sulfhydryl (SH) groups contents were determined according to Diao et al. (2016), measuring the absorbances at 370 nm and 412 nm, respectively. The values were expressed as nmol carbonyl/mg protein and nmol SH/mg protein using extinction coefficients of 22,000 M^−1^ cm^−1^ and 13,600 M^−1^ cm^−1^, accordingly. The surface protein concentration (Γ) was calculated from the equation: Γ(mg/m^2^) = M D[3,2]/6 V, where: M, protein mass adsorbed at interface; V, volume of rapeseed oil.

### Antioxidant capacity

The solutions for: ABTS^•+^, DPPH^•^ and FRAP (ferric reducing/antioxidant power) assays were prepared according to Serpen et al. (2012) with small modification. The radical-cation ABTS^•+^ was produced by the reaction between ABTS (aqueous solution) and potassium persulfate (PPE) to receive final concentration of 7 mM ABTS and 2.45 mM PPE. The mixture was then kept in dark at room temperature (20 °C) for 24 h. The ethanolic DPPH^•^ stock solution (0.1 mM) was prepared daily and stored (4 °C, dark) in a flask covered with aluminum foil. Both ABTS^•+^ and DPPH^•^ solutions before the usage were diluted with ethanol to an absorbance of 0.70 ± 0.02, measured at 734 nm and 517 nm, respectively. FRAP solution was prepared by diluting an aqueous solution of 10 mM TPPZ and 20 mM ferric chloride in 300 mM sodium acetate buffer (pH 3.6) at a ratio of 1:1:10 (v:v:v). MBSs were diluted with distilled water in the proportion 1:5 (v/v) and then the samples (0.5 mL) were mixed with 7.5 mL of: ABTS^•+^, DPPH^•^ or FRAP working solutions, incubated (20 °C, 20 min, dark) and centrifuged (2400 g, 10 min). The absorbance of supernatants at: 734, 517 and 593 nm in relation to ABTS, DPPH and FRAP methods, respectively was measured against a blank. Antioxidant capacity (AC) of MBSs was expressed in mM trolox equivalent (TE) using a trolox calibration curve.

### Sensory analysis

Sensory analysis of MBSs was performed at room temperature (22 °C) using an acceptance test with 55 untrained tasters after: 1, 10, 30, 60 and 90 days of cold storage. For the acceptability of: color, odor, consistency and firmness, a nine-point structured hedonic scale was used that ranged from one (strongly disliked) to nine (strongly liked). The results were transformed into desirability values (d_i_) according to procedures described by Espitia et al. (2014). The desirability index (DI) was calculated using the equation: DI = (d_1_ × d_2_ × … d_a_)^1/a^, where: d_1_, d_2_, etc. are individual desirability values of different attributes and a, number of attributes. The DI values were interpreted as the lowest (0) and highest (1) desirability.

### Statistical analysis

All experiments were performed in triplicate and are reported as the mean ± standard deviation. Tukey′s test was used to determine significant (*p* < 0.05) differences between means. The effects of extract type (ET) and storage time (ST) on studied parameters were analyzed by a two-way analysis of variance (ANOVA). Correlation coefficients (r) were determined using Pearson′s correlation. The analyses were carried out using Statistica 8.0 software (StatSoft Inc., USA).

## Results and discussion

### Antioxidant capacity

Three different assays (ABTS, DPPH, FRAP) were used to study antioxidant capacity (AC) of meat-based sauces (MBSs) and the values obtained are presented in Table 1. Compared to control sample (CS), addition of extracts (GCE, GTE, KRE), significantly (*p* < 0.05) increased AC of MBSs and this was observed regardless of the applied method and storage time (ST). The outcome of ANOVA revealed that the ability of antioxidants to quench ABTS^•+^ radicals was mainly dependent on extract type, ET [F = 143.7, *p* < 0.001], whereas inactivation of DPPH^•^ and reduction of Fe^3+^ to Fe^2+^ (FRAP) were predominantly affected by ST: [F = 126.8, *p* < 0.001] and [F = 102.7, *p* < 0.001], respectively. In relation to applied extracts, the AC magnitudes, demonstrated differentiated descending trends with increasing storage time (ST), plausible due to the various kinetics of phenolic compounds (PCs) oxidation, promoted by the increasing concentration of free radicals (Fan et al. 2017; Roginsky and Alegria 2005; Wildermuth et al. 2016). Generally, higher trolox equivalent (TE) values were found for the ABTS assay than DPPH and FRAP. This can be interpreted in terms that ABTS^•+^ is soluble in aqueous and organic solvents, therefore ABTS assay is more applicable to evaluate antioxidants with different hydrophobicity than DPPH^•^ and FRAP methods which generally are restricted to polar matrices (Gülçin 2010; Serpen et al. 2012). Taking into account TE magnitudes in relation to ET, they can be generally arranged in following order: GTE > GCE > KRE. This probably was associated with the chemical structure of PCs (catechins, chlorogenic acids, resveratrol) in the extracts, particularly with the number of included hydroxyl groups, their strength and positions (ortho, meta, para) on the hydrocarbon ring. Moreover, ABTS^•+^ is mainly inactivated by HAT mechanism, DPPH^•^ simultaneously by HAT and SET mechanisms, whereas the reduction of Fe^3+^ to Fe^2+^ is included in the SET mechanism (Liang and Kitts 2014). Hence, it would appear reasonable to speculate that various magnitudes of thermodynamic parameters, i.e. bond dissociation enthalpy of phenolic groups and ionization potential contributed to the observed efficacy of PCs (Labidi et al. 2018).

**Table 1:** Antioxidant capacity expressed as trolox equivalent (TE) of meat-based sauces assessed by: ABTS, DPPH and FRAP methods in relation to extract type (GCE, GTE, KRE) and storage time

Assay	Extract	Storage time (days)				
		1	10	30	60	90
	CS*	0.73 ± 0.04bA	0.71 ± 0.08b A	0.64 ± 0.06bA	0.46 ± 0.07aA	0.42 ± 0.03aA
ABTS (mM TE)	GCE	3.54 ± 0.16cB	3.28 ± 0.14bcB	2.94 ± 0.13abB	2.83 ± 0.11aC	2.75 ± 0.13aC
	GTE	4.89 ± 0.18cD	4.79 ± 0.19cD	4.48 ± 0.15cD	3.84 ± 0.12bD	3.29 ± 0.11aD
	KRE	3.86 ± 0.12cC	3.75 ± 0.12cC	3.27 ± 0.13bC	2.28 ± 0.08aB	2.25 ± 0.07aB
	CS*	0.54 ± 0.04cA	0.49 ± 0.07cA	0.42 ± 0.05bA	0.39 ± 0.06bA	0.28 ± 0.03aA
DPPH (mM TE)	GCE	2.97 ± 0.14cB	2.56 ± 0.11bB	2.35 ± 0.12abB	2.29 ± 0.10aB	2.11 ± 0.09aC
	GTE	3.46 ± 0.13cC	3.28 ± 0.12cC	2.71 ± 0.10bC	2.43 ± 0.09abC	2.29 ± 0.11aD
	KRE	2.84 ± 0.11cB	2.63 ± 0.13cB	2.27 ± 0.09bB	2.13 ± 0.11abB	1.98 ± 0.08aB
	CS*	0.49 ± 0.06cA	0.42 ± 0.04bcA	0.36 ± 0.05bA	0.27 ± 0.03aA	0.23 ± 0.04aA
FRAP (mM TE)	GCE	2.52 ± 0.11cB	2.19 ± 0.09bB	2.05 ± 0.12abB	1.94 ± 0.08aC	1.79 ± 0.07aC
	GTE	2.69 ± 0.10cCB	2.43 ± 0.08bC	2.11 ± 0.07aB	1.96 ± 0.09aC	1.87 ± 0.10aC
	KRE	2.74 ± 0.11cC	2.58 ± 0.09cC	2.27 ± 0.08bC	1.81 ± 0.07aB	1.68 ± 0.06aB

^*^Control sample. Values followed by different letters within the same row (a–c) and column (A–D), separately for: ABTS, DPPH and FRAP, differ significantly (*p* < 0.05). For the definition of abbreviations see Sect. 2

### Free fatty acids, lipid oxidation

Changes in the content of free fatty acids (FFA) are shown in Table 2. ANOVA revealed that formation of FFA was mainly affected by ST [F = 124.6, *p* < 0.001]. Irrespectively of the applied extracts, the quantities of FFA demonstrated ascending trend (r ≥ 0.993, *p* < 0.001) with increasing ST, and after 90 days of storage were found to be in order: GTE > CS > GCE > KRE. This could be interpreted on the grounds that liberation of FFA as the effect of hydrolysis of ester bonds in lipids was driven by both heating (manufacturing process) and differentiated physical stability of MBSs that they exhibited during ST (Vidrih et al. 2010). Moreover, this process could also be catalyzed by lipases, which are usually found at the lipid-water interface and are very active in the hydrolysis of triacylglycerols. The differences found between studied samples, supplemented with different extracts, were most likely associated with various degrees of FFA peroxidation. As reflected by ANOVA, peroxide values (PVs) were mainly affected by ST [F = 117.2, *p* < 0.001]. In general, the PVs demonstrated increasing trend with ST (r ≥ 0.889, *p* < 0.05) and only significant (*p* < 0.05) decrease was observed between 60 and 90 days of storage, regarding sample prepared with KRE (Table 2). The formation of primary oxidation products most probably resulted from the processing conditions (grinding, emulsification, heating) and slowly developing autoxidation mechanism accelerated by the increasing FFA content (Cheng 2016). However, it should be emphasized, that apart from CS, the PVs determined concerning MBSs, regardless of ST, were lower than the upper limit, 10 mEq O_2_/kg lipids (Codex Alimentarius 2001). Basically, this is in line with Galanakis (2018) research who reported that addition of olive phenols to the meat product retarded fats deterioration and ultimately extended the shelf-life. The found results can be attributed to the fact that proteins, adsorbed at the oil–water interface, interacted with starch molecules and both these biopolymers were also involved in interactions with PCs. Thus, it is possible to suppose that the polylayered interfacial coatings well protected lipid phase against diffusion of radicals from the external to internal phase of emulsion (Fan et al. 2017; Yildirim-Elikoglu and Erdem 2017). TBARS content was mainly dependent on extract type, ET [F = 119.4, *p* < 0.01] moreover, with the exception of samples enriched with GTE also well correlated with ST (r ≥ 0.909, *p* < 0.05). Compared to CS, extract addition in majority decreased TBARS concentration, particularly (*p* < 0.05) regarding samples stored for 60 and 90 days (Table 2). Similar trend associated with the addition of GTE to meat emulsion, was reported by Jongberg et al. (2015). After 90 days of storage, TBARS (mg MDA/kg lipids) were found to be in order: 2.15 (GTE) < 4.19 (GCE) < 5.21 (KRE) < 8.23 (CS). Excluding CS, the TBARS values were lower than 8 mg MDA/kg oil what may suggest good quality of the fat phase (Jiménez-Martín et al. 2015).

**Table 2:** Effects of storage time of meat-based sauces and extracts addition (GCE, GTE, KRE) on the free fatty acids (FFA) content, peroxide value (PV) and formation of thiobarbituric acid reactive substances (TBARS)

Parameter	Extract	Storage time (days)				
		1	10	30	60	90
	CS*	0.11 ± 0.02aA	0.14 ± 0.02aA	0.26 ± 0.04bA	0.45 ± 0.02cA	0.61 ± 0.03 dB
FFA (oleic acid, %)	GCE	0.12 ± 0.02aA	0.19 ± 0.02bB	0.31 ± 0.02cB	0.42 ± 0.03dA	0.57 ± 0.03eA
	GTE	0.09 ± 0.01aA	0.13 ± 0.01aA	0.24 ± 0.04bA	0.49 ± 0.02cB	0.68 ± 0.06 dB
	KRE	0.14 ± 0.01aA	0.15 ± 0.03aA	0.29 ± 0.03bB	0.43 ± 0.04cA	0.54 ± 0.04dA
	CS*	4.26 ± 0.21aC	6.12 ± 0.26bD	8.49 ± 0.36cC	9.73 ± 0.57dD	12.9 ± 0.94eC
PV (mEq O_2_/kg lipids)	GCE	4.31 ± 0.19aC	4.98 ± 0.13bB	6.53 ± 0.28cB	7.79 ± 0.19 dB	7.35 ± 0.31 dB
	GTE	3.28 ± 0.16aA	4.09 ± 0.11bA	5.07 ± 0.12cA	6.57 ± 0.21dA	6.21 ± 0.29dA
	KRE	3.84 ± 0.11aB	5.43 ± 0.29bC	6.11 ± 0.25cB	8.62 ± 0.37eC	7.96 ± 0.42 dB
	CS*	1.76 ± 0.14aC	3.12 ± 0.27bD	4.69 ± 0.38cC	6.17 ± 0.46dD	8.23 ± 0.53eD
TBARS (mg MDA/kg lipids)	GCE	1.83 ± 0.09aC	2.54 ± 0.26bC	3.51 ± 0.25cB	3.85 ± 0.19 dB	4.19 ± 0.32eB
	GTE	0.62 ± 0.07aA	1.13 ± 0.14bA	1.98 ± 0.16cA	2.46 ± 0.18eA	2.15 ± 0.17dA
	KRE	1.34 ± 0.12aB	2.01 ± 0.16bB	4.25 ± 0.31cC	5.12 ± 0.29dC	5.21 ± 0.35dC

^*^Control sample. Values followed by different letters within the same row (a–e) and column (A–D), separately for FFA, PV and TBARS, differ significantly (*p* < 0.05). For the definition of abbreviations see Sect. 2

### Carbonyls, total sulfhydryl content, surface protein concentration

ANOVA revealed that generation of protein carbonyls was predominantly affected by ST [F = 134.2, *p* < 0.001]. In all studied sets of samples carbonyls content gradually increased with raising ST. However it is noteworthy, that after 60 days of storage the magnitudes of this parameter were significantly (*p* < 0.05) lower concerning all samples fortified with extracts than those regarding CS (Table 3). These results are partly consistent with those obtained by other authors. For example Cao and Xiong (2015) showed that chlorogenic acid inhibited carbonyl formation regarding oxidatively stressed myofibrillar proteins, similar behavior was reported also by Zhang et al. (2018) in relation to epigallocatechin-3-gallate. The explanation of found results could be that growing with ST carbonyls content was driven by meat processing, during which, the integral structure of protein cells was destroyed, which contributed to their susceptibility to oxidation. Carbonyls content was also well correlated with TBARS (r ≥ 0.927, *p* < 0.05). Similar relationships found Al-Hijazeen et al. (2016) concerning cooked chicken breast meat during storage. Therefore, it is possible to speculate that binding of non-protein carbonyl compounds from lipid peroxidation to protein amino acid side chains, also contributed to proteins oxidation. According to the ANOVA, changes in total sulfhydryl groups (SH) content were mostly dependent on ST [F = 173.9, *p* < 0.001]. In all studied sets of samples values of this parameter demonstrated descending tendency with ST, and during 90 days of storage SH content in comparison to initial value decreased by: 45.7% and 42.1%, 51.3%, 47.5% regarding CS and MBSs enriched with: GCE, GTE, KRE, respectively (Table 3). Decrease in SH groups in systems containing PCs was reported in relation to: porcine myofibrillar protein containing chlorogenic acid (Cao and Xiong 2015); meat emulsions fortified with GTE (Jongberg et al. 2015); pork myofibrillar protein with the addition of catechin (Jia et al. 2017). The progressively lowering SH quantity with ST may be interpreted in terms that probably PCs promoted unfolding of protein structure and consequently induced conversion of SH groups to disulfide (S–S). Moreover, PCs could be oxidized to the corresponding quinones and formed covalent thiol–quinone adducts (TQ-adducts) (Diao et al. 2016; Jiang et al. 2018; Jongberg et al. 2015). The differences in SH content found after 90 days of storage may be also considered taking into account the fact that some of the PCs were able to substitute more than one SH group. The surface protein concentration (Γ) is presented in Table 3. ANOVA revealed that values of this parameter were predominantly affected by ST [F = 83.4, *p* < 0.001]. In all studied sets of samples Γ magnitudes continuously decreased with ST and the differences calculated between 1 and 90 days of storage exhibited the following order: GTE (0.63 mg/m^2^) > CS (0.48 mg/m^2^) > GCE (0.38 mg/m^2^) > KRE (0.32 mg/m^2^). This could be associated with partial replacement of proteins from the oil–water interface by small molecular surfactants, e.g. phenolic compounds or mono- and diacylglycerols formed by the triacylglycerols hydrolysis. Moreover, complexation with PCs could modify adsorption behavior of proteins and thus affect their surface concentration (Karefyllakis et al. 2017).

**Table 3:** Effects of storage time of meat-based sauces and extracts addition (GCE, GTE, KRE) on the protein oxidation (carbonyl content, sulfhydryl content) and surface protein concentration (Γ)

Parameter	Extract	Storage time (days)				
		1	10	30	60	90
Carbonyl (nmol/mg protein)	CS*	1.23 ± 0.09aB	2.07 ± 0.12bC	2.94 ± 0.17cC	3.98 ± 0.21dD	5.13 ± 0.29eD
	GCE	1.08 ± 0.06aB	1.96 ± 0.17bC	2.21 ± 0.13bB	3.57 ± 0.16cC	3.82 ± 0.18cB
	GTE	0.83 ± 0.03aA	1.14 ± 0.09bA	1.85 ± 0.06cA	2.68 ± 0.12dA	2.94 ± 0.13eA
	KRE	1.46 ± 0.11aC	1.72 ± 0.08bB	2.87 ± 0.14cC	3.24 ± 0.15 dB	4.29 ± 0.27eC
Sulfhydryl (nmol/mg protein)	CS*	25.4 ± 1.02eB	23.2 ± 0.61 dB	19.8 ± 0.64cC	17.9 ± 0.79bC	13.8 ± 0.42aC
	GCE	24.7 ± 0.87eB	21.9 ± 0.97 dB	18.3 ± 0.76cB	16.5 ± 0.72bB	14.3 ± 0.63aC
	GTE	22.4 ± 0.69eA	19.6 ± 0.74dA	15.2 ± 0.51cA	13.7 ± 0.49bA	10.9 ± 0.75aA
	KRE	23.6 ± 0.61eA	20.7 ± 0.82dA	17.6 ± 0.84cB	15.3 ± 0.83bB	12.4 ± 0.58aB
Γ (mg/m^2^)	CS*	1.62 ± 0.04aA	1.53 ± 0.06aA	1.27 ± 0.03bA	1.19 ± 0.04cA	1.14 ± 0.05cB
	GCE	1.61 ± 0.07aA	1.47 ± 0.09aA	1.36 ± 0.06bB	1.28 ± 0.04cB	1.23 ± 0.06cC
	GTE	1.65 ± 0.09aA	1.52 ± 0.04bA	1.34 ± 0.05bB	1.16 ± 0.03cA	1.02 ± 0.08dA
	KRE	1.59 ± 0.04aA	1.46 ± 0.04bA	1.38 ± 0.03cB	1.32 ± 0.06cB	1.27 ± 0.09cC

^*^Control sample. Values followed by different letters within the same row (a–e) and column (A–D), separately for: carbonyl, sulfhydryl and Γ, differ significantly (*p* < 0.05). For the definition of abbreviations see Sect. 2

### Rheological properties

**Fig. 1:**
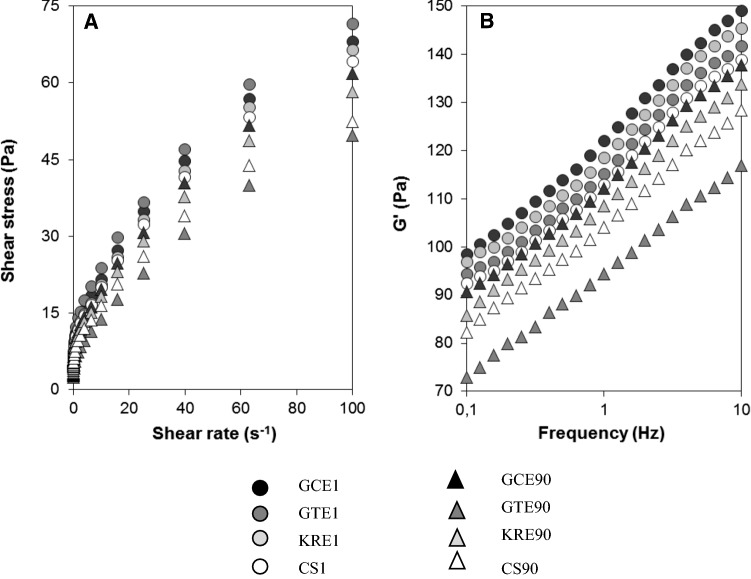
Shear stress versus shear rate (**a**) and storage modulus (G′) versus frequency (**b**) plots for meat-based sauces, depending on the extract type (GCE, GTE, KRE) and storage time (1 and 90 days). CS, control sample. For the definition of abbreviations see Sect. 2

The steady rheological measurements showed, that all examined samples of MBSs behaved as non-Newtonian fluids and demonstrated shear-thinning (pseudoplastic) behavior with yield stress (σ_0_). The flow curves determined after one day (CS1, GCE1, GTE1, KRE1) and 90 days of storage (CS90, GCE90, GTE90, KRE90) are shown in Fig. 1a. The increase in shear stress (decrease in apparent viscosity) can be interpreted that with raising shear rate the aggregated particles, and entangled polymers were progressively deformed, and aligned in the direction of flow. Oscillatory tests were performed because they are sensitive to changes in composition and structure of food emulsions. Studies revealed, that in majority MBSs exhibited typical behavior of rigid gels with storage modulus (G′) values higher than the loss modulus (G″) throughout the entire examined frequency range (data not shown). In Fig. 1b are presented values of G′ regarding MBSs stored for one day (CS1, GCE1, GTE1, KRE1) and 90 days (CS90, GCE90, GTE90, KRE90). Loss tangent (tan δ) was taken to express the ratio of G′′ to G′. In general, the values of tan δ demonstrated ascending tendency with raising frequency and ST, presumably due to the increasing destruction of the continuous gel phase. Considering frequency of 1 Hz and 1–90 days ST, the tan δ values were in ranges: 0.16–0.29 and 0.15–0.21, 0.17–0.36, 0.14–0.23, regarding CS and MBSs enriched with: GCE, GTE, KRE, respectively. The greater than 0.1 values of tan δ revealed the liquid-like feature of MBSs. Experimental data of steady shear rheology were fitted to the Herschel–Bulkley′s model and the obtained values of the coefficients are presented in Table 4. ANOVA demonstrated that σ_0_ values were mainly affected by ST [F = 69.2, *p* < 0.001]. In all tested sets of samples the magnitudes of σ_0_, decreased with ST. Compared to CS, magnitudes of σ_0_ after 90 days of storage, were higher by 21.6% and 10.9% regarding samples prepared with GCE and KRE, respectively and lower by 35.4% concerning sample fortified with GTE. The presence of a certain value of σ_0_ is very desirable in products with emulsion structure, because particles can be remained suspended in the medium. This parameter contributes to the resistance to flow at low deformations and may favor kinetic stability of dispersed systems. Moreover, reasonable magnitude of σ_0_ is of outmost importance in many industrial processes such as spreading or coating, and is strongly accepted by the consumers because, emulsions can keep original structure and adhere to solid components of salads (Bortnowska et al. 2014). Similarly as σ_0_, the values of consistency coefficient (k) demonstrated decreasing trend with ST and according to ANOVA were mainly dependent on ST [F = 74.8, *p* < 0.001]. The differences in k magnitudes, determined between initial values and those detected after 90 days of storage were found to be in order: GTE (2.07 Pa s^n^) > CS (0.75 Pa s^n^) > KRE (0.66 Pa s^n^) > GCE (0.65 Pa s^n^). These results show that with increasing ST the viscous nature of MBSs was decreased, due to raise of fluidity. The values of flow behavior index (n) were in ranges: 0.562–0.609 and 0.558–0.572, 0.552–0.634, 0.559–0.584, concerning CS and MBSs supplemented with: GCE, GTE, KRE, respectively (data not shown). The n values indicate the degree of shear-thinning behavior, and can be interpreted in terms that with increasing ST the pseudoplasticity of MBSs decreased (Bortnowska et al. 2013). Data from the dynamic rheological tests were fitted to the Bohlin′s model and thus obtained proportional coefficient (A_B_) and coordination number (z) are presented in Table 4. According to the ANOVA, A_B_ and z values were mostly influenced by ET [F = 82.3, *p* < 0.001] and ST [F = 69.3, *p* < 0.001], respectively. Both these parameters showed declining tendency with ST, what could be attributed to the fact that the number of flow units interacting with each other (z) and interaction strength between the flow rheological units (A_B_) decreased (Bortnowska et al. 2014). In complex systems such as MBSs rheological properties are dependent on interactions between all components. The interactions between protein and starch molecules are mainly electrostatic in nature, and occur between the anionic groups of the starch and the positively charged groups of the protein (Zhu 2015). Moreover, both starch and proteins may interact with PCs. The mechanisms of proteins involve covalent (reaction of o-quinones with nucleophilic groups of proteins such as –NH_2_ and –SH) or/and non-covalent interactions (hydrogen bonds, hydrophobic interactions, van der Waals attractions), leading to irreversible or/and reversible bindings, respectively (Yildirim-Elikoglu and Erdem 2017; Jiang et al. 2018). Whereas, the interactions of starch with PCs can be considered in terms that PCs consist of one or multi-hydroxyl groups that can interact both covalently and non-covalently, forming starch-phenolic complexes (Chi et al. 2019; Zhu 2015). Higher values (compared to CS) of rheological parameters, obtained from Herschel–Bulkley′s (k, σ_0_) and Bohlin′s (A_B_, z) relations, found concerning MBSs fortified with GCE or KRE may be related to the fact that PCs of these extracts, also oxidized to quinones, interacted with macromolecules (proteins, starch) and acted as their crosslinkers (Cao and Xiong 2015; Jongberg et al. 2013). The lower values (compared to samples enriched with GCE or KRE) of rheological parameters regarding MBSs prepared with GTE, may suggest loss of the intermolecular interactions (Karunaratne and Zhu 2016). The detrimental effect of PCs addition on the rheological properties has been reported also by other researchers (Chi et al. 2019; Jia et al. 2017; Li et al. 2018; von Staszewski et al. 2014). A plausible explanation for this behavior is that phenolic compounds of GTE attenuated starch hydrogen bonding network and covering proteins and starch molecules surfaces, shielded the intermolecular interactions (Cao and Xiong 2015; Jongberg et al. 2015).

**Table 4:** Herschel–Bulkley’s parameters (yield stress, σ_0_; consistency coefficient, k), Bohlin’s parameters (proportional coefficient, A_B_; coordination number, z) and desirability index (DI) of meat-based sauces, depending on the extract type (GCE, GTE, KRE) and storage time

Parameter	Extract	Storage time (days)				
		1	10	30	60	90
σ_0_ (Pa)	CS*	5.39 ± 0.14dA	5.12 ± 0.16cA	4.96 ± 0.13cA	4.63 ± 0.17bA	4.35 ± 0.11aB
	GCE	5.67 ± 0.11bB	5.56 ± 0.19bB	5.32 ± 0.14aB	5.17 ± 0.14aB	5.29 ± 0.18aD
	GTE	5.95 ± 0.12dC	5.71 ± 0.21 dB	5.34 ± 0.11cB	4.52 ± 0.16bA	2.81 ± 0.14aA
	KRE	5.49 ± 0.10cA	5.48 ± 0.13cB	5.42 ± 0.16cB	5.36 ± 0.12bB	4.82 ± 0.15aC
k (Pa s^n^)	CS*	4.27 ± 0.15cA	4.11 ± 0.07cA	3.93 ± 0.11bA	3.68 ± 0.17aA	3.52 ± 0.12aB
	GCE	4.78 ± 0.11bB	4.56 ± 0.16bC	4.32 ± 0.13aB	4.19 ± 0.15aB	4.13 ± 0.17aC
	GTE	5.04 ± 0.17dC	4.89 ± 0.21dD	4.48 ± 0.19cB	3.76 ± 0.13bA	2.97 ± 0.11aA
	KRE	4.57 ± 0.16bB	4.29 ± 0.11aB	4.16 ± 0.14aB	4.07 ± 0.09aB	3.91 ± 0.19aC
A_B_ (Pa s^1/z^)	CS*	114 ± 1.79dA	109 ± 2.39cA	105 ± 1.78bA	104 ± 3.02bB	99 ± 2.53aA
	GCE	125 ± 1.87cC	123 ± 2.68cC	120 ± 2.64bC	119 ± 2.14bD	114 ± 2.76aC
	GTE	118 ± 2.36cB	111 ± 2.23bA	109 ± 2.76bB	98.9 ± 2.38aA	95.7 ± 2.89aA
	KRE	121 ± 2.29bB	116 ± 2.45aB	114 ± 2.85aB	113 ± 2.71aC	108 ± 1.78aB
z (–)	CS*	9.58 ± 0.13bA	9.37 ± 0.13aA	9.26 ± 0.11aA	9.18 ± 0.16aA	9.12 ± 0.19aA
	GCE	9.93 ± 0.09bB	9.84 ± 0.14bB	9.78 ± 0.17bC	9.76 ± 0.15bB	9.56 ± 0.06aB
	GTE	9.82 ± 0.12cB	9.79 ± 0.09cB	9.53 ± 0.08bB	9.14 ± 0.07aA	8.98 ± 0.12aA
	KRE	9.91 ± 0.14cB	9.82 ± 0.15bB	9.75 ± 0.12bC	9.67 ± 0.13bB	9.46 ± 0.09aB
DI	CS*	0.43 ± 0.03bA	0.41 ± 0.01bA	0.38 ± 0.02bA	0.32 ± 0.03aA	0.28 ± 0.02aA
	GCE	0.67 ± 0.04aC	0.65 ± 0.05aC	0.63 ± 0.04aC	0.62 ± 0.04aC	0.61 ± 0.04aD
	GTE	0.66 ± 0.05cC	0.64 ± 0.04cC	0.61 ± 0.03cC	0.41 ± 0.02bB	0.34 ± 0.03aB
	KRE	0.49 ± 0.02bB	0.48 ± 0.02bB	0.46 ± 0.05bB	0.45 ± 0.03bB	0.41 ± 0.02aC

^*^Control sample. Values followed by different letters within the same row (a–d) and column (A–D), separately for: σ_0_, k, A_B_, z and DI, differ significantly (*p* < 0.05). For the definition of abbreviations see Sect. 2

### Stability and color

**Fig. 2:**
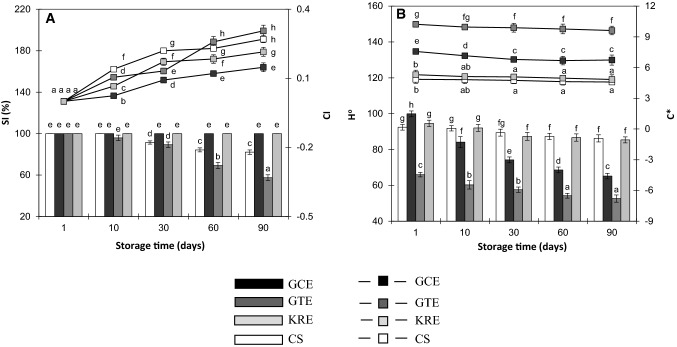
Stability index (SI, columns), coalescence index (CI, lines) (**a**) and hue angle (H°, columns), chroma (C*, lines) (**b**) of meat-based sauces enriched with: GCE, GTE, KRE. CS, control sample. Mean values, within the same characteristics, marked with no common letters are significantly different (*p* < 0.05). For the definition of abbreviations see Sect. 2

The values of stability index (SI), coalescence index (CI) and hue angle (H°), chroma (C*) during cold storage of MBSs are shown in Fig. 2a and b, respectively. Physical stability (PS) is a key quality parameter of emulsion sauces, therefore it should be determined, especially in the case of changes in emulsion composition (Bortnowska et al. 2013). Visual observation revealed that there was no phase separation in any of the samples kept at 4 °C in dark up to period of 90 days. However, differences in PS were found when the MBSs were subjected to centrifugation assay. Samples fortified with GCE and KRE demonstrated 100% stability during the whole ST, whereas CS and MBS containing GTE were stable (100%) until 10 days of storage and then SI magnitudes decreased (CS, r = –0.967, *p* < 0.01; GTE, r = –0.994, *p* < 0.001), reaching final values after 90 days of 82.1% (CS) and 57.5% (sample with GTE) (Fig. 2a). Coalescence index (CI) was determined to evaluate changes in droplet diameter during ST (Fig. 2a). In all studied sets of samples CI magnitudes were positively correlated with ST, however significant correlations were found only regarding MBSs containing PCs (r ≥ 0.891, *p* < 0.05). The outcome of ANOVA revealed that the CI quantities were affected by both ST = 142.3, *p* < 0.001] and ET = 67.9, *p* < 0.001]. After 90 days of storage, the values of CI (in relation to used extracts) can be arranged in the following order: GTE > CS > KRE > GCE. Karefyllakis et al. (2017) reported that physical interactions between sunflower proteins and chlorogenic acid resulted in a decrease in interfacial tension, consequently oil droplets exhibited high stability against coalescence. However, opposite effects were reported by von Staszewski et al. (2014) regarding interactions between β-lactoglobulin and green tea polyphenols (GTPs). It has been suggested that GTPs components get stacked to hydrophobic side chains of the amino acids in such a way that their hydrophobic domains could not be fully available to penetrate the oil–water interface. It can be therefore deduced, that in studied MBSs the extent of coalescence was largely dependent on interactions between PCs and proteins, due to the changes in their surface hydrophobicity, and thus surface activity (Cao and Xiong 2015). Moreover, reduction in surface protein concentration (Table 3) and changes in rheological properties (Table 4) should be considered as well. Differences in color of MBSs during ST were examined in relation to hue angle (H°) and chroma (C*). According to ANOVA, the values of H° were mainly dependent on ET = 126.1, *p* < 0.001]. Irrespectively of ET the quantities of H° demonstrated declining trend with increasing ST (r ≤  − 0.887, *p* < 0.05). Considering 1–90 days of ST, the values of H° were in ranges: 92.4–86.2 (CS), 99.8–65.2 (GCE), 65.9–52.6 (GTE) and 94.5–85.4 (KRE) (Fig. 2b). H° represents the degree of: redness, yellowness, greenness and blueness and this shows that color of MBSs changed with ST as follows: CS (green yellow–slightly yellow, GCE (green–green yellow), GTE (green yellow–slightly orange), and KRE (green yellow–slightly yellow). Comparing to initial value, the chroma values (C*) during 90 days of storage decreased by: 5.36% and 11.2%, 5.98%, 8.71% in relation to CS and MBSs enriched with: GCE, GTE, KRE, respectively (Fig. 2b). ANOVA yielded more significant effect of ET = 117.3, *p* < 0.001] than ST [F = 5.32, *p* < 0.01] on the C* values. Chroma values denote color purity or saturation and higher values indicate more vivid color. This may suggest that the colorfulness of the MBSs decreased with ST, irrespectively of the added extracts. Changes in color of MBSs during ST probably were associated with oxidative and non-oxidative reactions of phenolic compounds, resulting in colored condensation products (De Souza et al. 2017; Wildermuth et al. 2016).

### Desirability index

Desirability index (DI) was used to express hedonic acceptability of MBSs (Table 4). The values of DI were correlated negatively with ST as follows: CS (r =  − 0.998, *p* < 0.001), GCE (r =  − 0.934, *p* < 0.05), GTE (r =  − 0.975, *p* < 0.01) and KRE (r =  − 0.981, *p* < 0.01). The greatest decrease (48.5%) of DI (in comparison to initial value) during 90 days of storage was found concerning MBSs prepared with GTE most probably due to the worsening of physical stability (Fig. 2a) and rheological properties (Table 4). From opposite reasons assumptively, the lowest decline of DI (8.94%) was observed concerning samples prepared with GCE. At each time point, DI magnitudes regarding CS and samples fortified with KRE were significantly lower (*p* < 0.05) compared to MBSs containing GCE, most probably due to smaller chroma values (Fig. 2b). It has to be also underlined that after 90 days of storage slight unpleasant odor (related to rancidity) was reported only in control sample (data not shown).

## Conclusion

Physical stability (PS) of meat-based sauces (MBSs) was predominantly dependent on extract type (ET). No changes during the whole cold storage time (ST) were observed regarding samples enriched with KRE and GCE, whereas those fortified with GTE decreased stability after 10 days of storage. Fresh prepared MBSs demonstrated in majority higher values of rheological parameters (σ_0_, k, A_B_, z) than those found in control sample (CS), which with increasing ST decreased, particularly noticeably in samples prepared with GTE. Lipid markers (FFA, PV, TBARS) showed ascending trend with ST, however after 90 days their values were in majority smaller concerning MBSs prepared with phenolic extracts than those related to CS. Both ST and extracts addition differentiated process of protein oxidation and the lowest and highest increase of protein carbonyls was found in GTE enriched sample and CS, respectively. Descending trend with raising ST was observed concerning surface protein concentration and sulfhydryl content. Irrespectively on the used assay and ET, the values of antioxidant capacity of MBSs, were higher concerning samples prepared with phenolic extracts than those found in CS. Sensory assessment revealed that the highest value of desirability index, after 90 days of storage, was determined concerning sample enriched with GCE, whereas the lowest one regarding CS. From practical viewpoint, these results can have important implications for the development of commercial meat-based sauces with well-protected chemically labile components, exhibiting a relatively high level of antioxidant capacity throughout the 90 days of cold storage.
